# Detection of serum neutralizing antibodies to Simbu sero-group viruses in cattle in Tanzania

**DOI:** 10.1186/s12917-015-0526-2

**Published:** 2015-08-15

**Authors:** Coletha Mathew, S. Klevar, A. R. W. Elbers, W. H. M. van der Poel, P. D. Kirkland, J. Godfroid, R. H. Mdegela, G. Mwamengele, M. Stokstad

**Affiliations:** Department of Production Animal Clinical Sciences, Norwegian University of Life Science, P.O. Box 8146, Dep 0033 Oslo, Norway; Norwegian Veterinary Institute, Oslo, Norway; Central Veterinary Institute, Wageningen University and Research Centre, Lelystad, The Netherlands; Elizabeth McArthur Virology Laboratory, Narellen, Australia; University of Tromsø, Tromsø, Norway; Sokoine University of Agriculture, Morogoro, Tanzania

**Keywords:** Antibody ELISA, *Orthobunyavirus*, Serology, Schmallenberg virus, Virus neutralizing test

## Abstract

**Background:**

Orthobunyaviruses belonging to the Simbu sero-group occur worldwide, including the newly recognized Schmallenberg virus (SBV) in Europe. These viruses cause congenital malformations and reproductive losses in ruminants. Information on the presence of these viruses in Africa is scarce and the origin of SBV is unknown. The aim of this study was to investigate the presence of antibodies against SBV and closely related viruses in cattle in Tanzania, and their possible association with reproductive disorders.

**Results:**

In a cross-sectional study, serum from 659 cattle from 202 herds collected in 2012/2013 were analyzed using a commercial kit for SBV ELISA, and 61 % were positive. Univariable logistic regression revealed significant association between ELISA seropositivity and reproductive disorders (OR = 1.9). Sera from the same area collected in 2008/2009, before the SBV epidemic in Europe, were also tested and 71 (54.6 %) of 130 were positive. To interpret the ELISA results, SBV virus neutralization test (VNT) was performed on 110 sera collected in 2012/2013, of which 51 % were positive. Of 71 sera from 2008/2009, 21 % were positive. To investigate potential cross reactivity with related viruses, 45 sera from 2012/2013 that were positive in SBV ELISA were analyzed in VNTs for Aino, Akabane, Douglas, Peaton, Sabo, SBV, Sathuperi, Shamonda, Simbu and Tinaroo viruses. All 45 sera were positive for one or more of these viruses. Twenty-nine sera (64.4 %) were positive for SBV, and one had the highest titer for this virus.

**Conclusions:**

This is the first indication that Aino, Akabane, Douglas, Peaton, Sabo, SBV, Sathuperi, Shamonda and Tinaroo viruses circulate and cause negative effect on reproductive performance in cattle in Tanzania. SBV or a closely related virus was present before the European epidemic. However, potential cross reactivity complicates the interpretation of serological studies in areas where several related viruses may circulate. Virus isolation and molecular characterization in cattle and/or vectors is recommended to further identify the viruses circulating in this region. However, isolation in cattle is difficult due to short viremic period of 2 to 6 days, and isolation in vectors does not necessarily reflect the situation in cattle.

## Introduction

Simbu sero-group viruses belong to the genus *Orthobunyavirus*, in the family *Bunyaviridae* and contain three RNA segments. These viruses are naturally capable of genetic reassortment, which can lead to development of new viral strains with altered biological properties. They are transmitted by arthropods, mainly biting midges from the genus Culicoides and mosquitoes [1]. Most cause sub-clinical infections in non-pregnant animals. In pregnant animals, some of these viruses readily cross the placenta causing fetal infections that are associated with abortion, premature birth, still birth and congenital abnormalities in calves, lamb, and kids. The abnormalities include arthrogryposis, porencephaly, hydrocephalus, cerebella hypoplasia and congenital hydranencephaly [2].

The Simbu sero-group includes Schmallenberg virus (SBV), a newly emerged livestock virus first identified in Germany in 2011 [3, 4]. Its genome has been found to be closely related to Douglas, Sathuperi and Shamonda viruses [5]. Full genome investigation has indicated that SBV belongs to the species *Sathuperi virus* and is a possible ancestor of the reassortant Shamonda virus [6]. The origin of SBV is unclear [5, 7].

A number of Simbu sero-group viruses have been found to be present in different parts of the world, including Africa, Asia, Australia and Israel [8–10]. They have been isolated from domestic and wild animals as well as from vectors. Akabane virus has been the most recognized virus in this group together with Shamonda and Aino virus [4]. Antibodies to Akabane virus have been found in cattle and sheep in Asia, the Middle East, Australia and Africa [11, 12]. Diseases associated with some Simbu sero-group viruses have been reported to occasionally cause significant economic losses in the Australian and Japanese livestock industries [12], while in Africa both viruses and their consequences are poorly reported. A disease with characteristic signs of SBV has been observed in cattle and sheep in South Africa and Zimbabwe [1].

In Africa, members of the Simbu sero-group have been isolated from *Culicoides* midges and domestic animals. These include Sabo, Sango, Sathuperi, Shamonda, Shuni, Simbu and Yaba viruses [13, 14]. Neutralizing antibodies to Akabane virus have been found in wild animals in different African countries south of the Sahara including Tanzania [15–17]. In Asia, Simbu sero-group viruses originally recognized in Africa have been recovered from cattle and *Culicoides* midges in 2004 [18, 19].

Diagnosis of infections caused by Simbu sero-group viruses has traditionally been accomplished by detection of specific antibodies using virus neutralization assays but more recently enzyme-linked immunosorbent assays (ELISA) have been used and some are available as commercially prepared kits. However, as these viruses were originally clustered on the basis of serological assays, extensive cross reactivity is often observed [20, 21]. Virus isolation can be achieved in cell cultures and direct virus detection and identification is possible using RT- PCR and other molecular detection methods. Subsequent sequencing of the genome gives more detailed information of the type of the virus infecting animals.

In Tanzania, little is known about the presence of Simbu sero-group viruses and their impact on reproduction in cattle. In addition, the origin and global geographic distribution of SBV is not known. Mixed farming and extensive management systems and the presence of wildlife protected areas may facilitate transmission of these viruses between wild and domestic animals. This environment is conducive for vector activity. The present study was therefore undertaken with the following aims:

1. To investigate the prevalence of SBV-antibodies in cattle in the southern highlands of Tanzania, using a commercially available indirect ELISA kits.
  o Investigate the SBV antibody prevalence before and after the epidemic in Europe.
  o Investigate the association between seropositivity and reproductive disorders.
2. To further analyze ELISA results using a series of VNTs for SBV and other Simbu sero-group viruses closely related to SBV.

## Materials and methods

### Study area

The present study was part of a larger project on infectious agents causing reproductive disorders in dairy cattle in Tanzania. It was conducted in four districts that included Mbarali district in Mbeya Region and Wanging`ombe, Njombe rural and Njombe urban districts in Njombe Region in the southern highlands of Tanzania between July 2012 and March 2013. The selected farms from Njombe and Mbeya regions practice two different grazing systems. Most of the cattle in Mbeya graze on pastures during the day and are kept indoors at night, while the majority of herds in Njombe are confined in cattle houses. However, cattle houses or barns in both locations are not sealed to vectors. Both dairy cattle, which are mixed breed (Holstein Friesian and Ayrshire with Tanzania short horn zebu), and pastoral local breed of cattle (zebu), were included.

### Study design and sampling

A cross-sectional study design was chosen. A total of 202 herds were included in the present study. Of these, 181 dairy herds were small scale with an average size of three cows per herd, two were medium scale dairy herds with about 20 cows each, one was a large scale dairy herd with about 350 cows and 18 were pastoral herds with an average of 15 cows per herd. Inclusion criteria for herds were to have one female above 6 months of age and that the farmer was willing to participate. All animals below 6 months of age were excluded. From herds with less than five cattle above 6 months, all were included in the study. From herds with more than five cattle, five were randomly selected. This made a total of 659 animals. In addition, serum samples from 130 cattle from the same area, collected in connection with another study during 2008–2009, were also included in the present study. Blood samples were collected once from each animal.

All herds were visited by qualified personnel and farmers were interviewed using a structured questionnaire. The questionnaire aimed to gather information about reproductive disorders in the herd and individual animals over the last 3 years. The questionnaire was designed to determine the presence or absence of abortions, delivery of weak/malformed calves, stillbirth, dystocia and retained fetal membranes. Abortion was defined as a cow giving birth to a calf at any stage of gestation before term. Stillbirth was defined as giving birth to a dead calf, dystocia was defined as any assisted birth and retained placenta when fetal membranes fail to expel 24 h after delivery. Farmers were informed on the study before commencement of data and sample collection and willingly agreed to participate.

### Sample treatment and antibody analysis

Blood samples (about 5 ml) were collected aseptically from the jugular vein in plain evacuated tubes. The samples were left at room temperature for about 12 h to allow serum separation, and then serum was decanted into sterile tubes and transported on ice to the local laboratory and immediately frozen at approximately −20 °C. Between laboratories samples were shipped to the destination laboratories on ice and then kept frozen at −20 °C until analysis.

### Indirect enzyme linked immunosorbent assay

All serum samples were analyzed at the Norwegian Veterinary Institute using a commercial ELISA kit (ID Screen^®^ Schmallenberg Virus Indirect multi-species screening test ID.Vet Innovative Diagnostics) according to the manufacturer’s instructions. For each sample the S/P percentage was calculated: S/P = (OD sample-OD negative control)/OD positive control-OD negative control ×100. Samples presenting S/P values less than or equal to 50 % were considered negative, S/P values between 50 and 60 % were considered doubtful and S/P values greater than 60 % were considered positive.

### SBV Virus Neutralization Test (VNT)

**Fig. 1:**
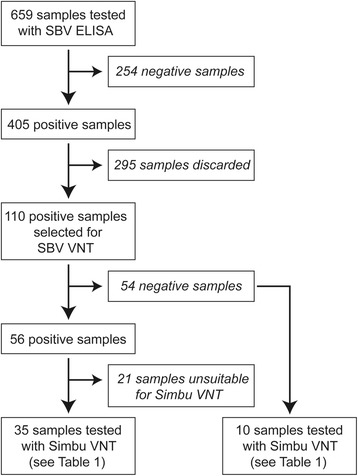
Overview of samples collected in 2012/2013 and used in different tests

The SBV VNT was performed on selected serum samples that tested positive in the SBV ELISA. This included 110 of 405 serum samples obtained in 2012/2013 (Fig. 1) and 71 of 130 sera collected in 2008/2009. Samples from 2012/ 2013 were selected to represent the whole study area. The test was performed at the Central Veterinary Institute, Lelystad, The Netherlands. In this test an SBV isolate from brain tissue of a lamb, fourth passage on Vero (African green monkey kidney) cells, was used. The VNT was performed on serum samples according to the method published in Loeffen et al. [22] with some small modifications: dilutions tested started at 1:4 and ended at 1:512. All samples were tested in duplicate. Titers were determined using the Reed-Muench method [23].

### VNT for other simbu sero-group viruses

SBV VNT positive (*n* = 35) and negative (*n* = 10) serum samples from 2012/2013 (Fig. 1) and 10 SBV ELISA positive (which were also positive in SBV VNT) and 10 negative serum samples from 2008/2009 were subjected to a VNT for Simbu sero-group viruses including Aino, Akabane, Douglas, Peaton, Sabo, SBV, Sathuperi, Shamonda, Simbu and Tinaroo at the Elizabeth MacArthur Agriculture Institute, Virology Laboratory, Menangle NSW Australia. Australian prototype strains of Aino, Akabane, Douglas, Peaton and Tinaroo viruses and prototype of Sabo, Sathuperi, Simbu and Shamonda viruses [20, 21] were used for these VNTs as described by Kirkland et al. [24] with the inclusion of the relevant virus in the test. For SBV the prototype strain originally isolated from Germany [4] was used. Selection of samples from 2008/2009 for this assay was based on availability of samples with enough volume.

### Data analysis

Data were analyzed using STATA version 12 for Windows (Stata Corp., College station, TX, USA) software. Positively infected animals were reported in proportions. The association between presence or absence of reproductive disorders and herd or individual animal serostatus (using SBV ELISA, SBV VNT and Simbu sero-group virus VNT) were analyzed using univariable logistic regression and reported as OR at 95 % CI, and level of significance at *P* < 0.05.

### Ethical statement

The protocol for field studies and collection of animal material was in accordance with ethical approval by the University Ethics Committee using guidelines from the Code of Conduct for Research Ethics of Sokoine University of Agriculture SUA/VET/012/04. Farmer’s verbal consent was sought before embarking on data and biological material collection.

## Results

### SBV antibody ELISA

Out of the 659 serum samples collected in 2012/2013, a total of 405 (61 %) were positive in the SBV antibody ELISA and out of 202 herds, 175 (87 %) had one or more seropositive animals. In Njombe out of 160 herds, 133 (83 %) were positive and out of 324 individual animals, 211 (65 %) were positive. In Mbarali however, all herds (*n* = 42) were positive while out of 335 individual animals, 194 (58 %) were positive. Seventy one (55 %) out of 130 sera collected in 2008/2009 were positive in the SBV ELISA. Antibodies were observed in sera collected from small, medium and large scale herds, Njombe and Mbarali, dairy and pastoral herds. The results of univariable regression analysis indicated no significant differences between serostatus and location or herd size or animal breed.

### SBV VNT

Fifty six (51 %) out of 110 serum samples collected in 2012/2013 were positive with titers ranging from 1:16 to 1:512 (Fig. 1). Fifteen (21 %) of 71 serum samples collected in 2008/2009 were positive in this assay with positive titers ranging from 1:16 to 1:768. Out of 71 samples from 2008/2009, 42 were toxic to the cell cultures which made it impossible to interpret the test results, while four were positive but with no end titers due to insufficient sample volume therefore their titers could not be determined.

### Other simbu sero-group virus VNTs

Antibodies against nine out of the ten Simbu sero-group viruses (Aino, Akabane, Douglas, Peaton, Sabo, SBV, Sathuperi, Shamonda and Tinaroo) were detected in one or more of the 45 serum samples from 2012/2013. Their titers ranged from 80 to 1280 (Table 1). Most animals had antibodies directed against two or more viruses. No antibody titers were detected against the species Simbu virus.

**Table 1:** Antibody titers to Simbu sero-group viruses detected in virus neutralizing test (VNT) performed on 45 cattle sera collected from Tanzania in 2012/2013

ID	AINO	AKABANE	DOUGLAS	PEATON	SABO	SBV	SATIV	SHAV	SIMBU	TINAROO	Highest titer
1	10	-	20	-	-	20	-	-	-	-	SBV/Douglas
2	20	-	40	40	-	20	-	-	-	-	Douglas/Peaton
3^a^	20	-	-	20	-	-	-	80	-	80	Tinaroo/SHAV
4	640	10	-	640	640	-	-	160	-	160	Aino/Peaton/Sabo
5	40	-	80	-	80	320	320	80	-	-	SBV/SATIV
6	10	-	-	20	-	-	-	-	-	40	Tinaroo
7^a^	-	-	40	-	-	-	-	-	-	-	Douglas
8^a^	40	10	40	80	≥1280	40	-	160	-	≥1280	Sabo/Tinaroo
9	20	-	-	640	160	-	-	-	-	-	Peaton
10	640	-	10	40	NT	10	NT	NT	NT	160	Aino
11	160	-	-	-	160	-	-	-	-	-	Aino/Sabo
12	20	-	20	160	-	10	80	-	-	160	Peaton/Tinaroo
13	80	-	-	20	80	-	-	-	-	80	Aino/Tinaroo/Sabo
14	-	-	-	-	80	-	-	160	-	160	Tinaroo/SHAV
15	320	-	-	-	-	-	-	-	-	80	Aino
16	-	-	-	-	80	80	160	80	-	-	SATIV
17	80	-	20	20	-	10	80	160	-	20	SHAV
18	160	-	20	40	-	40	80	320	-	80	SHAV
19	-	-	20	160	-	10	80	-	-	40	Peaton
20	160	-	-	-	-	-	80	160	-	10	Aino/SHAV
21	20	80	10	40	80	40	-	320	-	320	Tinaroo/SHAV
22	160	-	80	-	-	-	80	-	-	-	Aino
23	20	-	40	640	-	320	-	≥1280	-	40	SHAV
24	1280	-	20	160	160	10	80	160	-	20	Aino
25	20	-	20	80	80	20	160	320	-	80	SHAV
26	40	-	80	≥1280	-	160	160	-	-	-	Peaton
27	10	-	20	160	320	10	-	320	-	80	Sabo/SHAV
28	160	-	40	320	640	20	-	320	-	80	Sabo
29	40	-	80	-	-	160	-	-	-	-	SBV
30	80	10	-	320	160	-	-	320	-	160	Peaton/SHAV
31	40	-	20	160	-	20	-	80	-	160	Peaton/Tinaroo
32^a^	1280	-	40	1280	NT	40	NT	NT	NT	80	Peaton/Aino
33	10	-	160	-	≥1280	320	-	320	-	-	Sabo
34	1280	10	40	10	-	10	80	-	-	160	Aino
35^a^	20	20	20	160	320	40	-	320	-	≥1280	Tinaroo
36^a^	20	10	20	1280	80	40	-	640	-	320	Peaton
37	640	10	20	160	-	-	80	-	-	320	Aino
38	20	-	20	320	-	20	-	-	-	40	Peaton
39^a^	640	80	20	640	-	-	-	-	-	≥1280	Tinaroo
40^a^	80	-	80	-	-	160	-	640	-	160	SHAV
41	80	-	10	-	80	-	-	160	-	20	SHAV
42	≥1280	40	320	320	-	160	-	160	-	640	Aino
43^a^	80	-	-	80	320	-	-	160	-	80	Sabo
44^a^	≥1280	40	80	320	80	40	-	-	-	80	Aino
45	160	160	40	320	80	20	160	1280	-	≥1280	Tinaroo/SHAV

*SBV* schmallenberg virus, *SATIV* sathuperi virus, *SHAV* shamonda virus, *NT* not tested

^a^Samples negative in the first SBV VNT

Twenty nine (64.4 %) sera were positive for SBV, out of which seven had high titers (≥160) to this virus. One serum sample had highest titers for SBV, while two had the same titer to SBV and Douglas or Sathuperi virus. A high proportion (91.1 %) of the sera had antibodies against Aino virus and antibodies against Tinaroo, Douglas, Peaton, Shamonda, Sabo and Sathuperi were detected in 75.6, 73.3, 71.1, 55.6, 46.7 and 31.1 % of the animals respectively. There were 24 samples that had high titers to either Aino or Peaton viruses and five sera that had high titers only to Tinaroo virus. Eleven serum samples showed low antibody titers to Akabane virus while a single sample had a titer of 160. However all of these sera had very high titers to at least one of the other viruses (Table 1). None of the samples had positive titer to Simbu virus. Due to toxicity or bacterial contamination, sera collected in 2008/2009 were not suitable for this assay and were not included in the results.

### Association between reproductive disorders and serological results

Reproductive disorders were encountered in 104 out of 659 individual animals and they included either abortions, still birth, retained placenta, dystocia or calf malformations. There was a statistically significant association between the occurrence of one or more reproductive disorders and SBV ELISA seropositivity (OR = 1.9, 95 % CI = 1.2–2.9) on an individual animal level. There was no association demonstrated between individual animal seropositivity and any reproductive disorder alone. There was also no association observed between herd seropositivity and any specific reproductive disorder.

With the SBV VNT, there were no significant associations between any reproductive disorders and SBV seropositivity. Of the 45 sera subjected to Simbu sero-group VNT, eight originated from animals with a history of reproductive disorders. With Simbu sero-group VNT, there was only association between Akabane virus antibodies and abortion, and this association was not statistically significant (OR = 3.9, *P* = 0.059). Antibodies to none of the other viruses were associated with any of the reproductive disorders.

## Discussion

This is the first report of the presence of antibodies against viruses from the Simbu sero-group in cattle in Tanzania.

All animals tested with Simbu sero-group virus VNT had antibodies against one or more of the viruses. This is in line with the general view that Simbu sero-group viruses are endemic in Africa. There are reports of detection of antibodies to viruses like Shamonda, Sabo, Sango, Shuni, Igwavuma, Sathuperi and Akabane in the 1970s in Nigeria, South Africa and Kenya [13, 14, 25, 26]. However, due to the extensive cross reactivity that can occur between these viruses that are both genetically and antigenically closely related [14, 20, 27], it can be difficult if not impossible in some instances to determine with which virus(es) an animal has been infected.

Some animals had antibodies to only one virus, suggesting infection with the homologous or a very closely related virus. In other cases, there were very high titers to at least one virus and low titers to the others. Such patterns of reactivity could be interpreted as antibodies resulting from infection with one virus, with cross reactivity to other viruses. Alternatively, particularly for older animals, the low titers could indicate successive infections with different viruses in the Simbu sero-group. Dual or multiple infections in individual animals is also possible which would also complicate serological investigations.

It is possible that other viruses in the same group that were not included in the test are present. The occurrence of reassortants from two or more parental viruses can complicate the situation even further. It is known that some of these Simbu sero-group viruses are themselves reassortants. Phylogenetic studies have shown that Aino and Peaton viruses, Akabane and Tinaroo viruses and Shamonda and Sathuperi viruses are reassortants [7]. Phylogenetic analyses have also indicated that SBV is a reassortant with Sathuperi and Shamonda viruses, hence the possibility that an ancestor of SBV was created by co-infection with both viruses in the past [7]. However, in the context of this study, for interpretation of VNT results, only sharing or mobility of the M RNA segment is of relevance as it encodes the glycoproteins, against which the neutralising antibody response is developed. The ELISA kit used in the present study uses an entire recombinant N protein. There is extensive cross reactivity against N proteins as these are the group reactive antigens and also found in some of the reassortants [7].

Nevertheless, recognising these limitations in the interpretation of serological results, some broad trends can be deduced from the data. There is no evidence of infections with Simbu virus. There is little evidence of current infections with Akabane virus. Only a small proportion of animals were positive for Akabane virus and they had low antibody titers. Low titers may be due to declining antibody levels or could be caused by cross reactivity with other related viruses. Akabane has been shown to have cross reactivity with Shamonda, Sabo, Tinaroo and Yaba-7 viruses [20]. Our results support this finding since most of the samples with positive titers to Akabane also showed higher titers to Shamonda, Sabo or Tinaroo viruses. Therefore seropositivity to Akabane in our samples might be due to cross reactivity with the above viruses. As Simbu sero-group viruses are transmitted intermittently, it is possible that Akabane virus may not have been active in the 15-year period that the study animals represent (age data not shown). Akabane virus has been reported in wildlife in Tanzania in the past [16] and from neighboring Kenya [28]. Akabane virus has also been reported in cattle in many other African countries [26, 28–30] which shows that Akabane virus is endemic in Sub Saharan Africa that share ecological characteristics with parts of Tanzania.

Of the tested viruses, a large proportion of animals were seropositive to Aino, Peaton and Tinaroo viruses. Many of the positive animals also had very high antibody titers. Aino virus has only been reported previously in Japan and Australia [31, 32] not in Africa. This is also the first report of antibodies to Douglas virus, Peaton virus and Tinaroo virus in Africa and the first report of Sathuperi, Shamonda and Sabo viruses in Tanzania. Sathuperi, Shamonda and Sabo viruses have already been reported in Nigeria in 1970s [14] It is likely that these or very closely related viruses are each present as there were animals with very high antibody titers to a single virus and very low or no antibodies to the other viruses.

Antigenic relationships between Simbu sero-group viruses isolated in Africa and Australia have been documented. It is of particular interest to see how closely related these viruses are since both isolates have been used in the present study. Cross reactivity between Douglas and Sathuperi virus, Peaton and Sango virus and Tinaroo and Sabo viruses have been reported before [20]. However, in the present study there was no clear pattern of antibody levels to these virus pairs (Douglas/Sathuperi and Tinaroo/Sabo) being more associated with each other than to the other viruses. The close relationships between some of these viruses is reflected in their current taxonomic classification where the species Sathuperi virus includes Sathuperi, Douglas and Schmallenberg viruses; species Shamonda virus includes Shamonda, Peaton and Sango viruses; species Akabane virus includes Akabane, Tinaroo and Sabo viruses; species Shuni virus includes Aino and Shuni viruses and species Simbu virus includes only Simbu virus [6]. In the present study, the prevalence of positive animals to the traditionally Australian viruses Peaton, Tinaroo and Douglas viruses, was higher than to the Sabo, Sango and Sathuperi viruses that are traditionally African viruses. This suggests that cross reactivity is not the reason for the seropositivity to Australian strains in Tanzania, but rather that the animals are infected with these or other closely related unknown viruses. Among the positive animals, there was also no trend of higher titers to the African strains than the Australian strains.

Although virus neutralizing antibodies to SBV were detected in the 2012/2013 sera, there is no conclusive evidence that animals were infected with this virus. Most of the animals that were positive for SBV also had similar or higher titers in the VNT for one of the other Simbu sero-group viruses. Based on the interpretation that an animal is infected with the virus that gives the highest titre in a VNT, the results indicate the presence of SBV in Tanzania but only one animal had the highest titer to this virus. There are also some other possible explanations. Firstly, it might be that the animals have even higher titers for other viruses that were not included in the test assay, hence cross reactivity as discussed earlier. Secondly, the animals may not always produce the highest titers towards the infecting agent. In multiple infections especially with closely related agents, interpretation of the antibody titer may be a challenge [33]. Another study from Africa has reported antibodies against SBV in cattle and small ruminants in Mozambique [34]. However, the report is based only on results from tests with a commercial iELISA, which in the current study has been shown to lack specificity for SBV, and without confirmation with VNT or virus isolation. Disease with clinical presentation similar to SBV has also been reported in South Africa [17] but the clinical signs associated with SBV infections are not specific and no further confirmation of the diagnosis was undertaken.

Antibodies against SBV were also observed in the samples collected in 2008/2009, which if these are really SBV-specific antibodies, shows that it was probably already present in the area before the European epidemic. Further studies to confirm the presence of SBV in different African countries and compare its molecular characteristics with the European strains would be valuable. To definitively confirm if SBV is present or not, virus detection in the host will be necessary. The viremia induced by SBV is short lived, lasting for 2 to 6 days in cattle [5]. In the absence of an outbreak of congenital defects or clinical disease in mature animals, the chance of detecting the virus is very low. In the present study, 100 serum samples were tested in SBV RT-PCR, but all yielded negative results (results not shown). Surveillance in vectors could also be considered for virus detection and isolation which would allow further characterization using molecular methods. However, such studies will not prove that the virus is present in the cattle population. Nevertheless, this approach is most likely to unequivocally demonstrate the circulation of SBV in Africa.

The antibody ELISA indicated that many of the animals were positive for antibodies against SBV. However, a large proportion of ELISA positive samples were negative in the VNTs. As shown in table one, no SBV-specific antibody was detected in 16 out of 45 sera tested in multiple VNTs. A single sample that was negative in the SBV VNT had a low titre only to the closely related Douglas virus while the other 15 sera had neutralizing antibodies either to one other Simbu sero-group virus (two samples) or to multiple viruses (13 samples). The fact that several samples were negative to SBV and at the same time strongly positive for closely related viruses indicates that cross reactivity may not be the main reason for the seropositivity to SBV in the VNTs.

In general, commercially available ELISA assays are sensitive, specific and robust, but cross reactivity with other members of Simbu sero-group has been reported for the assay used in this study previously [35]. The initial validation of the assay was undertaken in Europe where other Simbu sero-group viruses have not been detected in cattle or sheep. As there is a high degree of similarity between the N protein antigens of members of the Simbu sero-group (the basis upon which these viruses were initially grouped), it is not surprising that cross reactivity is observed in the ELISA when animals are infected with one or more other Simbu sero-group viruses. Consequently, when interpreting the results of this and similar ELISAs, care must be exercised because a positive result may not indicate infection with SBV but could be due to infection with another Simbu sero-group virus. While VNTs are considered to be the ‘gold standard’ for the assessment of other assays, it is well recognised that even they are prone to cross reactivity for viruses belonging to the Simbu sero-group. As the ELISA is relatively easy to perform, requires minimal laboratory equipment, and laboratories do not need to have all reference viruses, it will be preferred in many of the regions where multiple Simbu sero-group viruses may be present. There is a need to validate ELISA kits for use in these endemic areas but this will be challenging due to the complex cross reactivity. Limited cross reactivity is even observed in monoclonal antibody based ELISAs run in either blocking or competitive formats (D.S. Finlaison and P.D. Kirkland, unpublished data). A goal of demonstrating specificity for the detection of antibodies to the Simbu sero-group should be the primary objective for an assay that is to be used for the confirmation of the cause of congenital defects in ruminants. Other virus specific assays such as real time PCR can then be used to undertake further testing of fetal specimens.

The present study showed an association between SBV ELISA positivity and reproductive disorders. As the ELISA is not SBV-specific, this reactivity may also be due to other Simbu sero-group viruses which are known to cause late abortion, premature birth, still birth and congenital malformations [3, 36, 37]. A clinical outbreak has not been reported in the study area. The lack of clinical outbreak could be due to endemic stability as it has been postulated for Akabane virus in Australia and for epizootic hemorrhage disease in white-tailed deer in the US [38]. However, the present study detected abortions but without any obvious fetal abnormalities. The reasonable interpretation of this result is that one or more of the Simbu sero-group viruses that are prevalent in the area result in a negative effect on reproductive performance. The syndrome of congenital defects associated with infection with Simbu sero-group viruses that has been reported in Japan, Australia and Israel has not been observed in Africa despite the presence and wide spread occurrence of antibodies to Simbu sero-group viruses in different animals [28].

Antibodies against Akabane virus were associated with abortion, however the association was not statistically significant and the serological evidence of the presence of Akabane virus is inconclusive. It is well known that Akabane virus causes abortion in bovine animals [2, 28]. The small sample size limits the power of the analysis of association between the different viruses included in VNTs and reproductive disorders.

## Conclusion

This is the first serological indication of Simbu sero-group viruses including SBV and their possible association with reproductive disorders in cattle in Tanzania. It is possible that viruses from the Simbu sero-group, other than the ones included in the test, are also present. The origin of the virus that caused the recent SBV epidemic in Europe is still a mystery but this study demonstrates the possibility that the virus may have been present in Tanzania already and other parts of Africa also where the same vectors are abundant. Isolation and further genetic characterization of the viruses, including isolates from different geographical origins, will be essential for understanding the molecular epidemiology and evolution of SBV related viruses.

## Abbreviations

SBV: Schmallenberg virus
SATIV: Sathuperi virus
SHAV: Shamonda virus
ELISA: Enzyme linked immunosorbent assay
VNT: Virus neutralization test
OR: Odds ratio
